# Continuing Professional Development ‐ Radiation Therapy

**DOI:** 10.1002/jmrs.799

**Published:** 2024-06-10

Maximise your CPD by reading the following selected article and answer the five questions. Please remember to self‐claim your CPD and retain your supporting evidence. Answers will be available via the QR code and published in JMRS – Volume 71, Issue 4 December 2024.

## Radiation Therapy ‐ Original Article

### The impact of a prophylactic skin dressing on surface‐guided patient positioning in chest wall Radiation Therapy

Cumming J , Thompson K , Woodford K , Panettieri V , Sapkaroski D . (2024) J Med Radiat Sci. 10.1002/jmrs.781 PMC1117704238525921

1. What is Mepitel?
  - A type of adhesive bandage used for wound dressing.
  - A brand of surgical gloves.
  - A software tool for medical record management.
  - A type of antimicrobial agent.
2. How can Mepitel film benefit patients receiving chest wall radiation therapy?
  - It helps visualise the treatment field.
  - It helps reduce adverse skin reactions by creating a protective barrier.
  - It creates a build‐up effect to get dose to the skin surface.
  - It covers the patient to help keep them warm.
3. Which of the following best describes how surface‐guided radiation therapy (SGRT) enhances radiation therapy treatments?
  - By utilising specialised imaging techniques to target tumours more accurately.
  - By reducing the duration of treatment sessions through faster delivery of radiation doses.
  - By delivering radiation from multiple angles simultaneously, increasing treatment precision.
  - By providing real‐time monitoring of patient motion and positioning during treatment.
4. According to the study findings, which of the following statistically significant differences were found in the setup accuracy of patients treated with Mepitel applied compared to those treated without?
  - Increased Online Corrections (OLC) magnitude in the Superior–Inferior axis, and overall Vector‐d measures only.
  - Increased OLC magnitude in all axes.
  - Decreased OLC magnitude in all axes.
  - No statistically significant differences were found.
5. According to the study findings, what conclusion can be drawn about Mepitel use in chest wall radiation therapy using SGRT for patient setup?
  - Mepitel creates clinically significant issues with setup accuracy when using SGRT and should not be used.
  - Mepitel does not have any impact on SGRT setup accuracy.
  - Mepitel is unlikely to have any clinical impact on SGRT setup accuracy.
  - No conclusion could be made from the data.

## Answers

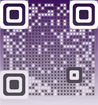

Scan this QR code to find the answers.
